# Supplementary material for: Asynchronous magnetic resonance elastography: Shear wave speed reconstruction using noise correlation of incoherent waves
Source: Magn Reson Med. 2022 Oct 27;89(3):990–1001. doi: 10.1002/mrm.29502 (PMC9792433; doi:10.1002/mrm.29502)
Supplement: Supplementary file 1 — DATA S1 MATLAB code used to generate the numerical simulations of Figure 1 [file MRM-89-990-s002.zip › k-Wave/helpfiles/makeMultiArc.html]

makeMultiArc :: Functions (k-Wave)


# makeMultiArc

Create a binary map of multiple arcs within a 2D grid.

## Syntax

```
[arcs, arcs_labelled] = makeMultiArc(grid_size, arc_pos, radius, diameter, focus_pos)
[arcs, arcs_labelled] = makeMultiArc(grid_size, arc_pos, radius, diameter, focus_pos, ...)
```

## Description

`makeMultiArc` creates a binary map of multiple arcs within a two-dimensional grid using `makeArc`. The position of the arcs is denoted by 1's in the matrix with 0's elsewhere. A labelled matrix can also be returned, where the position of the first arc is denoted by 1's, the position of the second arc by 2's, and so on.

## Examples

```
% define grid parameters
x_size              = 300e-3;
Nx                  = 256;
dx                  = x_size / Nx;
grid_size           = [Nx, Nx];

% create a Cartesian circle with the x, y positions of the arcs
circle_radius       = 125e-3;
num_arcs            = 16;
arc_pos             = makeCartCircle(circle_radius, num_arcs, [1, 1] * x_size / 2).';
 
% convert the Cartesian arc positions to grid points
arc_pos             = round(arc_pos/dx);

% define element parameters
radius              = round(x_size / (2 * dx));
diameter            = 21;
focus_pos           = [1, 1] * Nx/2;

% create arcs
makeMultiArc(grid_size, arc_pos, radius, diameter, focus_pos, 'Plot', true);
```

## Inputs

|  |  |
| --- | --- |
| `grid_size` | size of the 2D grid given as a two element vector [Nx, Ny] [grid points] |
| `arc_pos` | midpoint of each arc given as a matrix of dimensions N x 2, with each row specifying the midpoint of each arc as a two element vector [ax, ay] [grid points] |
| `radius` | radius of curvature of each arc given as either a single number (if the arcs have the same radius), or an N-element vector containing the radius for each arc [grid points] |
| `diameter` | diameter of each arc (length of straight line between the end points) given as either a single number (if the arcs have the same diameter), or an N-element vector containing the diameter for each arc [grid points] |
| `focus_pos` | any point on the beam axis of the arc given as either a two element vector (if the arcs have the same focus\_pos), or as a matrix of dimensions N x 2, with each row specifying the focus\_pos for each arc given as a two element vector [fx, fy] [grid points] |

## Optional Inputs

Optional 'string', value pairs that may be used to modify the default computational settings.

| Input | Valid Settings | Default | Description |
| --- | --- | --- | --- |
| `'Plot'` | *(Boolean scalar)* | `false` | Boolean controlling whether the arc array is plotted using `imagesc`. |

## Outputs

|  |  |
| --- | --- |
| `arc` | 2D binary map of an arc |
| `arcs_labelled` | 2D labelled matrix of arcs |

## See Also

`makeArc`, `makeMultiBowl`
